# Multiorgan failure caused by Chinese herbal medicine Xanthii Fructus poisoning: a case report

**DOI:** 10.1186/s12906-023-04105-6

**Published:** 2023-07-31

**Authors:** Yaqian Li, Guangcai Yu, Longke Shi, Liwen Zhao, Zixin Wen, Baotian Kan, Xiangdong Jian

**Affiliations:** 1 Department of Poisoning and Occupational Diseases, Emergency Medicine, Cheeloo College of Medicine, Qilu Hospital of Shandong University, Shandong University, Jinan, 250012 Shandong China; 2 School of Public Health, Cheeloo College of Medicine, Shandong University, Jinan, 250012 Shandong China; 3 School of Nursing and Rehabilitation, Cheeloo College of Medicine, Shandong University, Jinan, 250012 Shandong China; 4 Department of Geriatric Medicine, Qilu Hospital, Cheeloo College of Medicine, Shandong University, Jinan, 250012 Shandong China

**Keywords:** Xanthii Fructus, Liver function damage, Renal function damage, Multiorgan failure, Anuria, Jaundice, Case report

## Abstract

**Background:**

Xanthii Fructus was used in the treatment of rhinitis and related nasal disease. It is the most commonly used chemically active component in compounds formulated for the treatment of rhinitis. However, poisoning, resulting in serious consequences, can easily occur owing to cocklebur overdose, improper processing, or usage without processing.

**Case presentation:**

We reported on a 55-year-old man who experienced allergic rhinitis for 2.5 years. He ingested unprocessed Xanthii Fructus for 2 months as treatment. However, he developed anorexia; nausea; abdominal pain; general weakness; hiccups; oliguria and anuria; significantly elevated serum alanine aminotransferase, aspartate aminotransferase, and creatinine levels; and abnormalities in blood coagulation series. Nutritional support; daily drugs for liver protection, gastric protection, inflammation reduction; fresh plasma; and cryoprecipitate infusion were administered. Continuous venovenous hemodialysis (Prismaflex ST100) was also administered. However, the patient’s multiple organ failure gradually worsened, ultimately leading to death.

**Conclusion:**

Xanthii Fructus poisoning affects multiple systems, and its clinical manifestations are complex. Therefore, it is easily misdiagnosed and missed. Along with careful inquiry of medical and medication history, early diagnosis and intervention are vital for a successful treatment. It is also important to educate people and create awareness about this poisoning. Therefore, this intractable case has great clinical significance.

## Background

Xanthii Fructus (Latin binomial nomenclature: Xanthium sibiricum Patrin ex Widder), also known as Siberian Cocklebur. It is a common and well-known traditional Chinese herbal medicine, and it has been in use for thousands of years in China [1]. At present, the specific toxic components in Xanthii Fructus are not very clear, and the toxic chemical components mainly included water-soluble glycoside compounds and Poison protein. Because of its extensive biological and pharmacological activities, it is widely used to treat many diseases such as rhinitis, sinusitis, headache, gastric ulcer, urticaria, rheumatic bacteria, fungal infection, and arthritis [1, 2]. However, at higher doses, Xanthium is toxic, and it can damage multiple organs, especially the liver and kidney [3, 4]. To the best of our knowledge, although there are related reports on cases of Xanthium poisoning in animals [5], few reports on cases of caused by Xanthium poisoning in adults. This condition thus remains sufficiently uncommon, warranting further research. In this study, we present a case of multiple organ failure due to Xanthii Fructus poisoning, which, ultimately, led to death.

### Case presentation

A 55-year-old man experienced allergic rhinitis for 2.5 years with no hepatitis or kidney disease. He became ill at the sight of greasy food on January 12, 2022 and sought care at a local health center on January 13, 2022. The specific therapeutical program he received was unknown, and the effect was unhelpful. His daily urination frequency and volume gradually decreased. He also had epigastric pain, generalized weakness, and hiccups and was admitted to a local hospital on January 16, 2022. The laboratory test results were as follows: white blood cell (WBC) count, 8.95 × 10^9^/L (reference value 3.5–9.5 × 10^9^/L); platelets (PLTs), 9 × 10^9^/L (reference value 125–350 × 10^9^/L); direct bilirubin (DBIL), 135.07 µmol/L; indirect bilirubin (IBIL), 20.99 µmol/L; albumin (ALB), 36.5 g/L(reference value 40–55 g/L); alanine transaminase (ALT), 8,453 U/L (reference value 9–50 U/L); aspartate aminotransferase (AST), 3,515 U/L (reference value 15–45 U/L); serum ammonia (AMON) 435.79 µmol/L (reference value 18–72 µmol/L); creatinine (Cr), 809 µmol/L (reference value 40–115 µmol/L); blood urea nitrogen, 32.82 mmol/L(reference value 2.00–6.90 mmol/L); amylase (AMYL), 184 U/L(reference value 0–96 U/L); prothrombin time (PT), 55.1 s (reference value 8.80–13.80 mmol/L); and activated partial thromboplastin time (APTT), 64.50 s (reference value 26.00–42.00 mmol/L). No obvious abnormality was observed in the remaining parameters. The local hospital made a diagnosis of acute renal failure, acute liver failure, coagulation dysfunction. Considering the seriousness of the patient’s condition, he was transferred to the intensive care unit of the local city hospital. And symptomatic and supportive treatment was given (specific therapeutic schemes were unknown). After 5 days of treatment, the patient continued to experience abdominal pain, abdominal distention, and anuria. Transaminase levels decreased significantly, but serum creatinine and bilirubin levels remained unchanged. The laboratory test results after treatment were as follows: DBIL, 176.70 µmol/L; IBIL, 17.08 µmol/L; ALB, 35.1 g/L; ALT, 637 U/L; AST, 87 U/L; AMON, 47.79 µmol/L; Cr, 681 µmol/L (Table 1 shows the laboratory test results obtained in the local hospital).

**Table 1:** Laboratory results obtained in the local hospital and our hospital

Laboratory investigations	Normal range	1–16 8:55 (Local hospital)	1–16 13:40	1–17 6:22	1–18 6:03	1–19 5:27	1–20 1:42	1–21 7:30	1–21 18:59	1–22 14:05 (Our hospital)	1–24 8:36
WBC count (10 × 10^9^/L)	3.50–9.50	8.95	7.60	4.04	7.68	7.25	–	9.50	–	14.05	17.44
NEU# (10 × 10^9^/L)	1.80–6.30	8.23	7.00	3.75	7.01	7.01	–	9.00	–	13.65	16.52
LYM# (10 × 10^9^/L)	1.10–3.20	0.35	0.23	0.13	0.11	0.11	–	0.18	–	0.18	0.22
RBC (10 × 10^12^/L)	4.30–5.80	4.35	4.17	3.41	3.33	3.33	–	3.66	–	3.63	3.28
Hb (g/L)	130–175	142	137	111	108	108	–	122	–	118	108
PLT (10 × 10^9^/L)	125–350	89	98	77	66	66	–	70	–	57	56
ALT (U/L)	9–50	8453	8021	4405	1506	1506	1183	833	637	573	338
AST (U/L)	15–45	3515	3280	1510	232	232	103	84	87	138	101
DBIL (μmol/L)	0–7.64	135.07	127.80	103.70	104.20	104.20	113.48	132.20	176.70	297.00	233.60
IBIL (μmol/L)	0–15.00	20.99	24.72	16.21	17.38	17.38	23.47	30.93	17.08	86.5	94.1
ALB (g/L)	40–55	36.5	34.4	27.7	33.0	33.0	33	34.7	35.1	34	33.5
AMON (μmol/L)	18–72	435.79	359.16	256.64	131.85	131.85	97	79	47.79	59	28
Cr (μmol/L)	40–115	809	857	505	419	419	524	586	681	514	719
BUN (mmol/L)	2.00–6.90	32.82	34.61	20.90	20.66	20.66	30.20	36.31	41.83	42.80	45.40
BNP (pg/mL)	–	–	–	–	3870	3917	–	–	–	4718	24,049
UOP (mL)	1000–2000	20	0	0	0	0	0	0	0	0	0

*ALB* Albumin, *ALT* Alanine transaminase, *AST* Aspartate transaminase, *AMON* Ammonia, *BNP* NT-PROBNP, *BUN* Blood urea nitrogen, *CK* Creatine kinase, *Cr* Creatinine, *Hb* Hemoglobin, *LDH* Lactate dehydrogenase, *PLT* Platelet, *RBC* Red blood cell, *WBC* White blood cell, *CK-MB* Creatine kinase-MB, *hs-CTNI* High-sensitivity cardiac troponin I, *D-Di* D-Dimer, *MYO* Myoglobin, *IBIL* Indirect bilirubin, *DBIL* Direct bilirubin, *LYM#* Lymphocytes count, *NEU#* Neutrophils count, *AMYL* Amylase, *UOP* Urinary output

**Fig. 1:**
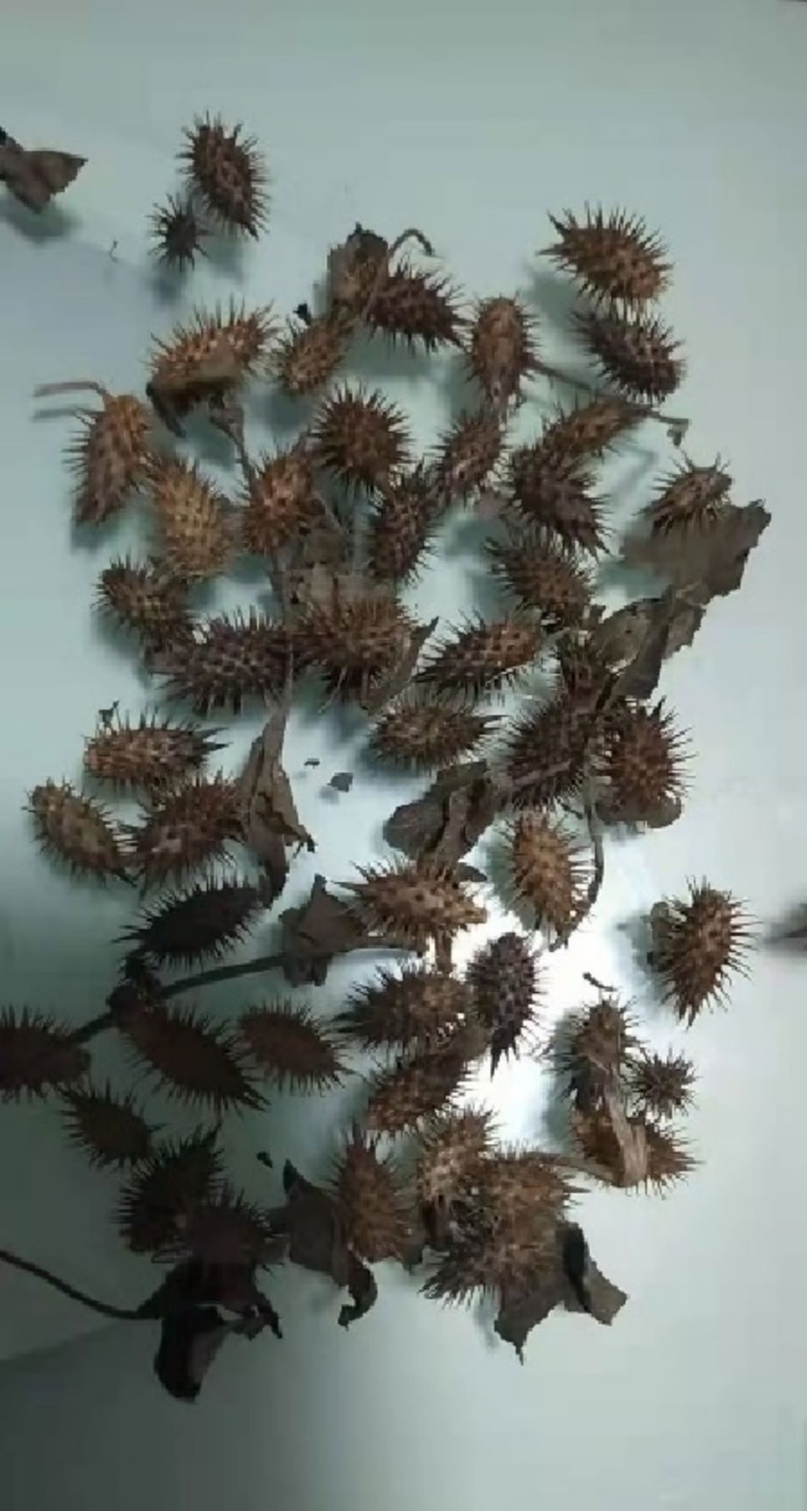
Image of the Xanthii Fructus taken orally by the patient

**Fig. 2:**
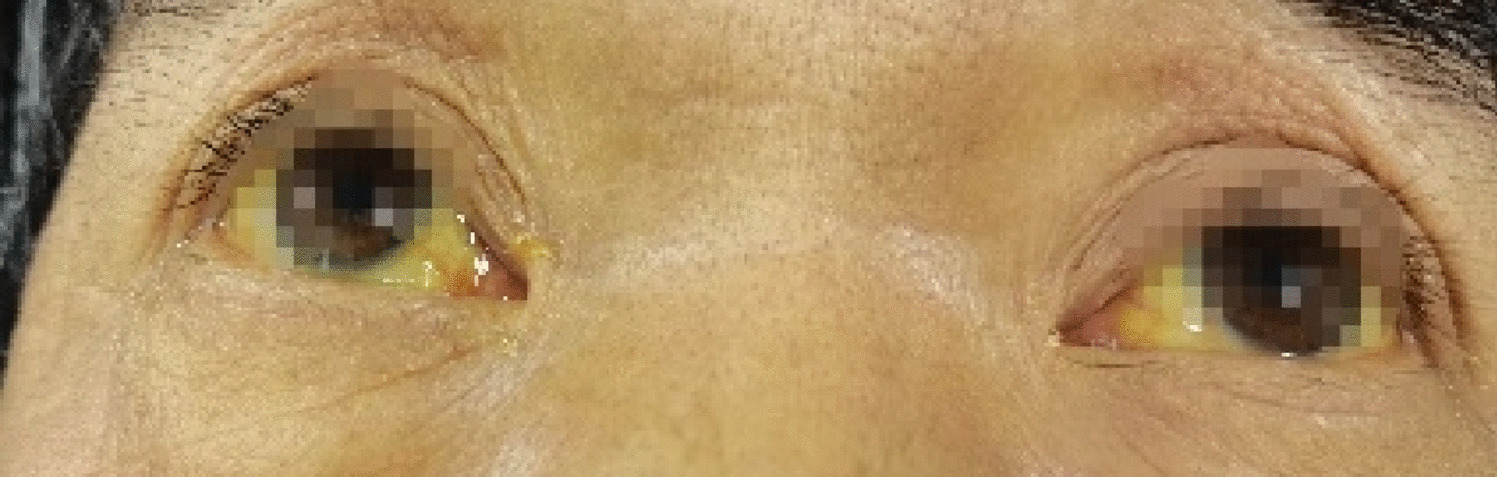
Image of the patient on being admitted to our hospital. The skin of the whole body and sclera had yellow coloration

**Fig. 3:**
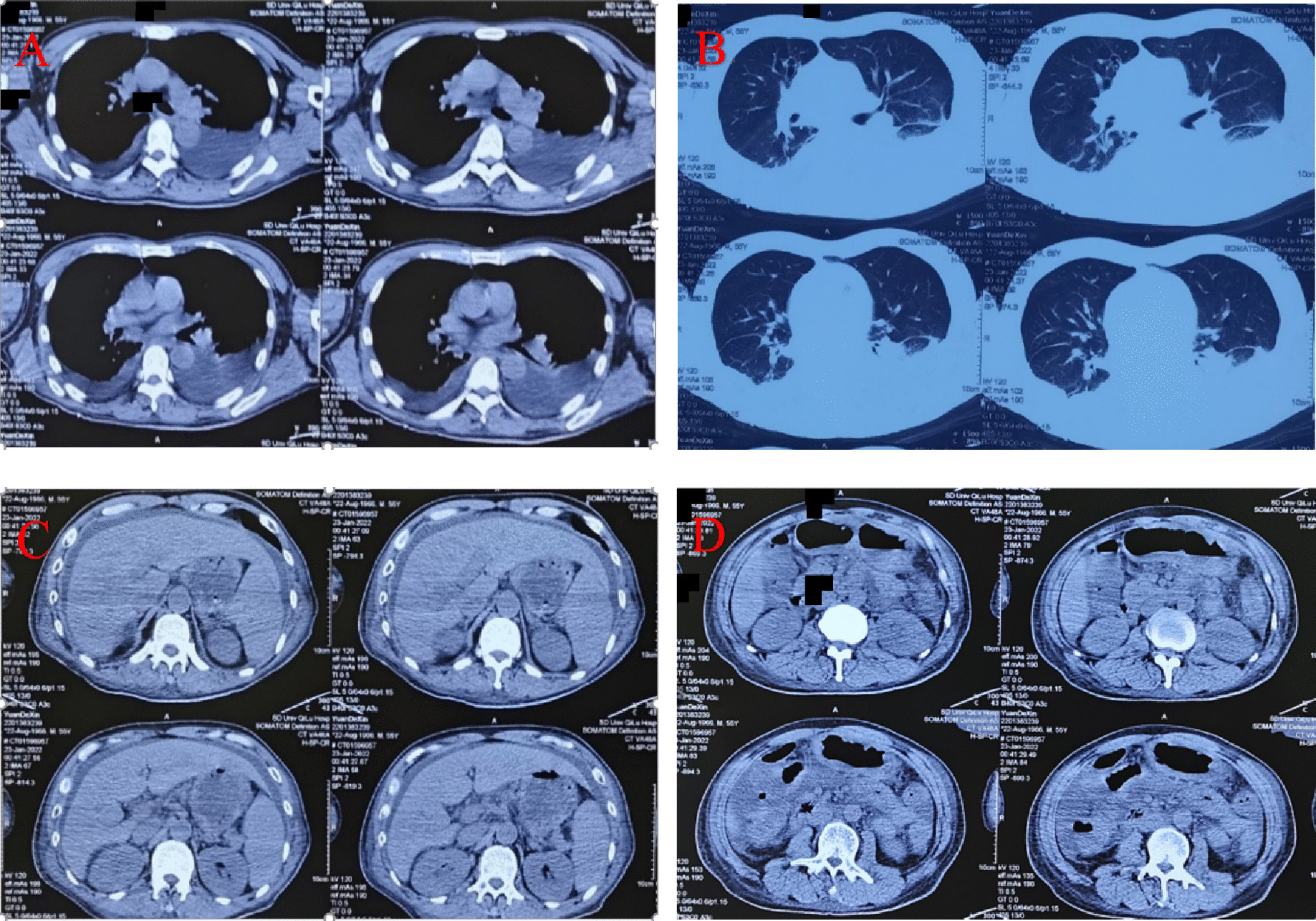
Chest computed tomography on admission showing inflammation of both lungs and manifestation of bilateral pleural effusion and adjacent lung tissue under expansion (**A** and **B**); Abdominal computed tomography on admission showing ascites and thickening of the gastric wall and part of the intestinal wall (**C** and **D**)

For further diagnosis and treatment, he was transferred to our department at on January 22, 2022. To determine the cause of the disease, the doctor repeatedly asked the patient’s history. It was then revealed that the patient had followed folk medication and consumed Xanthii Fructus (Fig. 1) for 2 months, approximately 20 g (35–40 particles), once daily (at night, before going to bed). During the same period, no other drugs were taken, and no toxic animals, plants, or chemicals were consumed. Upon admission to our hospital, his vital signs were as follows: temperature, 36.6 °C; heart rate, 79 beats/min; respiratory rate, 20 breaths/min; blood pressure, 131/75 mmHg; and oxygen saturation, 99%. He appeared to be in severe pain and severe yellowing of the skin and white of the eyes (Fig. 2). Heart and lung examinations showed normal findings. He also had a soft abdomen, tenderness of the upper abdomen, no rebound pain, and muscle tension, and the liver was palpable 2 cm under the rib margin. The spleen was not palpable under the ribs. Normal borborygmus was noted. Physiological reflexes were present, but no pathological reflexes, such as Babinski’s sign, were induced. The laboratory test results obtained in our department were as follows: WBC count, 14.05 × 10^9^/L; PLT, 57 × 10^9^/L; ALT, 573 U/L; AST, 138 U/L; AMON, 59 µmol/L; Cr, 514 µmol/L; serum myoglobin (MYO), 856.20 ng/mL (reference value 0–70 ng/mL), serum high-sensitivity troponin I (hs-CTNI), 22.70 ng/L; NT-PROBNP (BNP), 4,718 pg/mL; PT, 18.90 s, APTT, 43.90. No significant changes in electrocardiogram findings were observed. Chest computed tomography (Fig. 3A-B) revealed inflammation of both lungs and manifestations of bilateral pleural effusion and hypoexpansion of the adjacent lung tissues. Abdominal computed tomography (Fig. 3C-D) particularly revealed ascites and thickening of the gastric wall and part of the intestinal wall. Xanthii Fructus-related multiple organ dysfunction syndrome was diagnosed, and the treatment included nutritional support and daily administration of medications including lansoprazole (30 mg bid), magnesium isoglycyrrhizinate injection (Chinese Medicine Approval: H20051942, CTTQ, Lianyungang, Jiangsu Province, China) (0.2 g), furosemide (100 mg bid). Fresh plasma (200 mL) and cryoprecipitate (12 u) were also given. Continuous venovenous hemodialysis (Prismaflex ST100) was also administered.

On day 2, his heart rate was 146 beats/min, and blood pressure was 112/67 mmHg. The patient was irritable. He continuously had a stomachache, which did not improve, but, instead, gradually worsened. The patient was administered phloroglucinol (40 mg IM) for pain relief and esmolol micropump for heart rate control.

On day 3, the patient was delirious, drowsy and continued to have anuria. He developed sighing and open-mouth breathing. His blood pressure continued to drop to 38/21 mmHg, heart rate was 86 beats/min, and respiratory rate was 24 breaths/min, with 89% oxygen saturation. The laboratory test results were as follows: pH, 6.82; partial carbon dioxide pressure (PCO_2_), 64 mmHg; partial oxygen pressure (PO_2_), 277 mmHg; lactic acid (Lac), 7.80 mmol/L; K^+^, 7.5 mmol/L; WBC count, 17.44 × 10^9^/L; PLT count, 56 × 10^9^/L; DBIL, 233.6 µmol/L; IBIL, 94.1 µmol/L; ALB, 33.5 g/L; ALT, 338 U/L; AST, 101 U/L; Cr, 719 µmol/L; MYO, 6,114.00 ng/mL; hs-CTNI, 96.14 ng/L; BNP, 24,049 pg/mL; PT, 21.70 s; APTT, 43.50 s; Fib, 2.75 g/L; DD-i, 23.81 µg/mL. High-flow oxygen was administered, and IV norepinephrine was provided via micropump to increase blood pressure; additionally, the patient received an intravenous drip of nikethamide and lobeline to maintain breathing, intravenous drip of sodium bicarbonate to correct acidosis, and intravenous injection of calcium gluconate to reduce potassium levels. The pumping esmolol micropump was then stopped. After 30 min, the patient was still in coma (Glasgow Coma Scale score: 3 points), with blood pressure of 107/76 mmHg, heart rate of 113 beats/min, respiratory rate of 22 beats/min, and 100% oxygen saturation. Arterial blood gas analysis revealed the following: pH, 6.93; PCO_2_, 62 mmHg; PO_2_, 212 mmHg; Lac, 7.0 mmol/L; and K^+^, 6.90 mmol/L. The patient’s family gave up further treatment and arranged for discharge from the hospital. The patient died on the day of discharge (Table 1 shows the laboratory test results obtained in our hospital).

## Discussion and conclusions

It is well known that many factors affect the toxicity or nontoxicity of traditional Chinese medicine, including variety, processing method, compatibility, dosage form, decocting method, route of administration, dosage, and individual differences in physical characteristics [6]. If taken inappropriately, it can be very harmful and cause poisoning. As early as in the 1960s, Chinese scholars had presented cases of acute renal failure caused by excessive doses of the Chinese herbal medicine Mutong [7]. In 1993, Belgian researchers first presented cases of chronic progressive kidney failure caused by consumption of weight loss pills containing aristolochic acid [8]. In the twenty-first century, aconitine poisoning was also frequently reported [9].

The 2020 edition of Chinese Pharmacopoeia [10] classified Xanthii Fructus as a “toxic” medicinal compound. Toxicological studies showed that consumption of Xanthii Fructus and whole grass induced toxicity, and Xanthii Fructus was the most toxic compound [5]. The main toxic components of Xanthii Fructus and their mechanisms are as follows: 1) water-soluble glycoside compounds mainly including atractyloside and carboxyl atractyloside. Atractyloside and carboxyl atractyloside can inhibit the transport of ADP/ATP to proteins in vivo, leading to disorders related to acid–base balance metabolism and damage of liver and kidney function [11–13]. Obatomi et al. [12] reviewed their biochemical characteristics and poisoning mechanism in detail. 2) Poison protein: some scholars believe that the toxicity of Xanthii Fructus is due to its toxic protein. The toxicity of that protein can be reduced by soaking it in water or heating; for example, it can be denatured after being fried until brown and solidified in cells without being dissolved to achieve detoxification [14].

Hamza Karabiber et al. [15] described the case of a 15-year-old girl who developed fulminant liver failure and anuria because of eating approximately 80 seeds of Xanthii Fructus. Turgut et al. [16] also reported nine cases of carboxyatractyloside poisoning in humans whose initial symptoms occurred 3–48 h after ingestion. Luan Hong et al. [17] presented a case of acute liver and kidney failure caused by Xanthii Fructus whose initial symptoms occurred 20 days after ingestion. Mizael Machado et al. [5] presented a case of hepatic liver necrosis in ruminants and horses induced by oral consumption of Xanthii Fructus. After active treatment, serum transaminase levels were significantly reduced, and creatinine abnormality had not significantly changed.

At present, there is no specific antidote for Xanthii Fructus poisoning. Early detection and treatment can significantly increase the chances of survival for patients. Otherwise, symptomatic supportive therapy is the only treatment option [16]. Early oral Xanthii fruit poisoning can actively induce vomiting. Therefore, treatment should mainly focus on gastric lavage, liver protection, diuretics, and catharsis to promote toxin excretion, maintenance of water and electrolyte balance, and preservation of an antishock state. Ventilatory support should be provided if necessary. Antibiotic prevention and total parenteral nutrition can be considered for patients with severe pneumonia who are supported with a ventilator, and most critically ill patients are eventually treated with plasma exchange combined with hemodialysis [18].

In conclusion, we present a case of multiorgan failure due to Xanthii Fructus poisoning. This patient had consumed unprocessed Xanthium orally, once every night before going to bed, to treat allergic rhinitis, consuming a dosage of approximately 20 g/day (35–40 particles/day). After treatment, the patient’s condition continued to worsen, until finally, his family gave up treatment, and the patient died on the same day of discharge. Xanthii Fructus poisoning affects many systems, and its clinical manifestations are complex. Hence, it can easily be misdiagnosed or missed. Careful inquiry of medical and medication history, early diagnosis, and early intervention are keys to successful treatment. Additionally, it is particularly important to create awareness and educate people to prevent poisoning or even death caused by improper or accidental consumption of Xanthii Fructus.

## Data Availability

The datasets used during the current study available from the corresponding author on reasonable request.

## Abbreviations

ALB: Albumin
ALT: Alanine transaminase
AST: Aspartate transaminase
AMON: Ammonia
BNP: NT-PROBNP
BUN: Blood urea nitrogen
CK: Creatine kinase
Cr: Creatinine
Hb: Hemoglobin
LDH: Lactate dehydrogenase
PLT: Platelet
RBC: Red blood cell
WBC: White blood cell
CK-MB: Creatine kinase-MB
hs-CTNI: High-sensitivity cardiac troponin I
D-Di: D-Dimer
MYO: Myoglobin
IBIL: Indirect bilirubin
DBIL: Direct bilirubin
LYM#: Lymphocytes count
NEU#: Neutrophils count
AMYL: Amylase

## References

[1] Fan W, Fan L, Peng C, Zhang Q, Wang L, Li L (2019). Traditional uses, botany, phytochemistry, pharmacology, pharmacokinetics and toxicology of Xanthium strumarium L.: a review. Molecules.

[2] Nanjing University of Chinese medicine (2006). Dictionary of traditional Chinese medicine.

[3] Stuart BP, Cole RJ, Gosser HS (1981). Cocklebur (Xanthium strumarium, L. var. strumarium) intoxication in swine: review and redefinition of the toxic principle. Vet Pathol.

[4] Luciani S, Martini N, Santi R (1971). Effects of carboxyatractyloside a structural analogue of atractyloside on mitochondrial oxidative phosphorylation. Life Sci.

[5] Machado M, Queiroz CRR, Wilson TM, Sousa DER, Castro MB, Saravia A (2021). Endemic Xanthium strumarium poisoning in cattle in flooded areas of the Araguari River, Minas Gerais. Brazil Toxicon.

[6] Wang J, Wong YK, Liao F (2018). What has traditional Chinese medicine delivered for modern medicine?. Expert Rev Mol Med.

[7] Li X, Xia Y, Li G, Zhan Z, Yao R, Li M (2021). Traditional uses, phytochemistry, pharmacology, and toxicology of Akebiae Caulis and its synonyms: a review. J Ethnopharmacol.

[8] Ji H, Hu J, Zhang G, Song J, Zhou X, Guo D (2021). Aristolochic acid nephropathy: A scientometric analysis of literature published from 1971 to 2019. Medicine (Baltimore).

[9] Ya Y, Zhixiang Z, Chao L, Wei Z, Zhiyong W, Huafeng C (2021). Reflections on the aconitine poisoning. J Forensic Sci.

[10] National Pharmacopoeia Committee. Pharmacopoeia of the People’s Republic of China (volume one). Beijing: China Medical Science Press. p. 1088. 2020.

[11] Pebay-Peyroula E, Dahout-Gonzalez C, Kahn R, Trézéguet V, Lauquin GJ, Brandolin G (2003). Structure of mitochondrial ADP/ATP carrier in complex with carboxyatractyloside. Nature.

[12] Obatomi DK, Bach PH (1998). Biochemistry and toxicology of the diterpenoid glycoside atractyloside. Food Chem Toxicol.

[13] Xue LM, Zhang QY, Han P, Jiang YP, Yan RD, Wang Y (2014). Hepatotoxic constituents and toxicological mechanism of Xanthium strumarium L. fruits. J Ethnopharmacol.

[14] Qianfeng G. Processing methods of Chinese medicinal. Beijing: China Traditional Chinese Medicine Publishing House, 2016.08 Materials [M].

[15] Karabiber H, Almis H, Selimoglu MA, Yakinci C, Yilmaz S (2014). Xanthium strumarium poisoning requiring liver transplantation. J Pediatr Gastroenterol Nutr.

[16] Turgut M, Alhan CC, Gürgöze M, Kurt A, Doğan Y, Tekatli M (2005). Carboxyatractyloside poisoning in humans. Ann Trop Paediatr.

[17] Hong L, Xiao WC, Yan X (2019). A case of acute liver and kidney failure caused by Xanthii Fructus. J Clin N.

[18] Tingting Z, Liangchun Y, Junning Z (2010). Advances in studies on toxicity and modern toxicology research of Fructus Xanthii. Med Recapitulate.

